# High Thyrotropin Is Critical for Cardiac Electrical Remodeling and Arrhythmia Vulnerability in Hypothyroidism

**DOI:** 10.1089/thy.2018.0709

**Published:** 2019-07-17

**Authors:** Julieta Fernandez-Ruocco, Monica Gallego, Ainhoa Rodriguez-de-Yurre, Julian Zayas-Arrabal, Leyre Echeazarra, Amaia Alquiza, Victor Fernández-López, Juan M. Rodriguez-Robledo, Oscar Brito, Ygor Schleier, Marisa Sepulveda, Natalia F. Oshiyama, Martin Vila-Petroff, Rosana A. Bassani, Emiliano H. Medei, Oscar Casis

**Affiliations:** ^1^ Instituto de Biofísica Carlos Chagas Filho, Universidade Federal do Rio de Janeiro, Rio de Janerio, Brazil.; ^2^ Centro de Investigaciones Cardiovasculares, Conicet La Plata, Facultad de Ciencias Médicas, Universidad Nacional de La Plata, Buenos Aires, Argentina.; ^3^ Departamento de Fisiología, Facultad de Farmacia, Universidad del País Vasco UPV/EHU, Vitoria, Spain.; ^4^ National Institute of Cardiology (INC), Rio de Janeiro, Brazil.; ^5^ Center for Biomedical Engineering, University of Campinas, Campinas, Brazil.

**Keywords:** ionic currents, cardiac electrophysiology, repolarization, cardiomyocyte, thyroid

## Abstract

***Background:*** Hypothyroidism, the most common endocrine disease, induces cardiac electrical remodeling that creates a substrate for ventricular arrhythmias. Recent studies report that high thyrotropin (TSH) levels are related to cardiac electrical abnormalities and increased mortality rates. The aim of the present work was to investigate the direct effects of TSH on the heart and its possible causative role in the increased incidence of arrhythmia in hypothyroidism.

***Methods:*** A new rat model of central hypothyroidism (low TSH levels) was created and characterized together with the classical propylthiouracil-induced primary hypothyroidism model (high TSH levels). Electrocardiograms were recorded *in vivo*, and ionic currents were recorded from isolated ventricular myocytes *in vitro* by the patch-clamp technique. Protein and mRNA were measured by Western blot and quantitative reverse transcription polymerase chain reaction in rat and human cardiac myocytes. Adult human action potentials were simulated *in silico* to incorporate the experimentally observed changes.

***Results:*** Both primary and central hypothyroidism models increased the L-type Ca^2+^ current (I_Ca-L_) and decreased the ultra-rapid delayed rectifier K^+^ current (I_Kur_) densities. However, only primary but not central hypothyroidism showed electrocardiographic repolarization abnormalities and increased ventricular arrhythmia incidence during caffeine/dobutamine challenge. These changes were paralleled by a decrease in the density of the transient outward K^+^ current (I_to_) in cardiomyocytes from animals with primary but not central hypothyroidism. *In vitro* treatment with TSH for 24 hours enhanced isoproterenol-induced spontaneous activity in control ventricular cells and diminished I_to_ density in cardiomyocytes from control and central but not primary hypothyroidism animals. In human myocytes, TSH decreased the expression of *KCND3* and *KCNQ1*, I_to_, and the delayed rectifier K^+^ current (I_Ks_) encoding proteins in a protein kinase A–dependent way. Transposing the changes produced by hypothyroidism and TSH to a computer model of human ventricular action potential resulted in enhanced occurrence of early afterdepolarizations and arrhythmia mostly in primary hypothyroidism, especially under β-adrenergic stimulation.

***Conclusions:*** The results suggest that suppression of repolarizing K^+^ currents by TSH underlies most of the electrical remodeling observed in hypothyroidism. This work demonstrates that the activation of the TSH-receptor/protein kinase A pathway in the heart is responsible for the cardiac electrical remodeling and arrhythmia generation seen in hypothyroidism.

## Introduction

Recent studies highlight the importance of thyrotropin (TSH) levels in the mortality of both hypothyroid and euthyroid individuals. Patients with treated overt hypothyroidism with serum TSH levels between 0.5 and 5 mIU/L have significantly lower mortality rates than patients with TSH levels between 5 and 10 mIU/L (1). Moreover, a recent study demonstrated that high-normal TSH levels are associated with higher mortality than mid-normal levels in non-hypothyroid individuals (2), but the mechanisms responsible for this elevated mortality are unknown.

In this context, it has been reported that hypothyroid patients show electrocardiogram (ECG) abnormalities such as atrial fibrillation, prolonged QT and QTc intervals, and greater QT dispersion. This electrical remodeling creates a pro-arrhythmic substrate and facilitates the occurrence of ventricular arrhythmias including torsades de pointes (3–7). Surprisingly, patients with subclinical hypothyroidism, with normal triiodothyronine (T3)/thyroxine (T4) levels but elevated TSH, show similar repolarization alterations in the ECG as those with overt hypothyroidism (5,7), suggesting the involvement of TSH in cardiac electrical remodeling and arrhythmia. In the last decade, clinical observations have shown a direct correlation between serum TSH levels and QT/QTc prolongation in hypothyroid patients (4,5,7–9). In agreement with this hypothesis, it was recently reported that *in vitro* treatment of healthy cardiac myocytes with TSH decreases the amplitude of different repolarizing K^+^ currents and the expression of their channel proteins (10).

This study used animal models of primary (high TSH) and central (low TSH) hypothyroidism, as well as adult human atrial myocytes and computational modeling, to gain insight into the mechanisms underlying myocardial electrical remodeling by TSH. The results presented here suggest that the cardiac electrical remodeling and enhanced arrhythmia susceptibility in primary hypothyroidism under adrenergic stimulation are due to: (i) an increase of the depolarizing inward calcium current secondary to the reduction of thyroid hormone levels; (ii) a TSH receptor/protein kinase A (PKA)-dependent reduction of the expression of the pore-forming proteins of the channels that carry the repolarizing outward potassium currents I_to_ and I_Ks_; and (iii) the appearance of early afterdepolarizations (EAD) and arrhythmias secondary to prolongation of repolarization, especially under β-adrenergic stimulation. In summary, this work demonstrates the direct involvement of TSH in the cardiac electrical remodeling and arrhythmia predisposition seen in hypothyroidism.

## Methods

### Animals and induction of hypothyroidism

The investigation fulfilled the Spanish (RD 1201/2005), Brazilian (Federal Law 11794), and European (D2003/65/CE and R2007/526/CE) rules for animal care used for experimental and other research purposes, and has been approved by the Ethics Committee for Animal Care of the Universidad del País Vasco (CEBA/46/2010) and the Ethics Committee for Animal Care and Use of the University of Campinas (CEUA/B/UNICAMP, Brazil, No. 4429-1).

Hypothyroidism was induced in Sprague Dawley rats (200 g). Primary hypothyroidism was induced by the addition of 0.02% propylthiouracil (PTU; Sigma–Aldrich) to the drinking water for six weeks, which is a widely used, well characterized animal model of primary hypothyroidism. For induction of central hypothyroidism, animals were treated by daily gavage with bexarotene (BXT) for six weeks at a dose of 50 mg/kg, which was the lowest tested dose that induced hypothyroidism in 100% of the animals in preliminary experiments. Previous tests also showed an absence of differences between rats receiving daily gavage with dimethyl sulfoxide, which was used to dissolve BXT, and non-treated controls.

Animals were weighed weekly, and body temperature was taken as that measured from the outer ear with an infrared thermometer. Blood samples were collected from the tail vein under light anesthesia (with isofluorane), and hormone levels were determined with T4 and TSH enzyme-linked immunosorbent assay kits (Diametra and Demeditec Diagnosis Gmbh, respectively) following the manufacturers' instructions. After six weeks, the animals were killed by heart removal under deep anesthesia with ketamine and xylazine (50 mg/kg and 1.5 mg/kg, respectively, administered intraperitoneally [i.p.]). The thyroid glands were removed and immersed in 4% paraformaldehyde. After fixation, 30 μm sections were stained with hematoxylin and eosin.

### In vivo *electrophysiological experiments*

ECG recording was carried out in conscious animals with permanent surgical steel electrodes inserted under anesthesia, positioned in the DII lead, and connected by flexible cables to a differential amplifier MP35 (Biopac Systems, Inc.). The signal was filtered at 100 Hz and digitized at 1 kHz using BSL Pro v3.7 (Biopac Systems, Inc.). The measurements were performed blinded to the experimental groups. The QT interval was measured from the beginning of the QRS complex to the end of the T wave and was corrected for the heart rate using the Bazzet formula, QTc = QT/√R-R.

At the end of the six-week experimental period, the arrhythmia vulnerability test was performed in control and hypothyroid rats under isoflurane anesthesia. A baseline ECG was recorded for three minutes, and then rats were injected with caffeine (120 mg/kg i.p.) and dobutamine (50 μg/kg administered intravenously). The ECG was then recorded for 10 minutes, and the incidence of arrhythmias was quantified.

### Ventricular myocyte isolation

Cardiac myocytes were isolated in a Langendorff apparatus, as previously described (10,11). The hearts were mounted through the aorta on a Langendorff apparatus and perfused retrogradely with Tyrode solution containing (in mmol/L): NaCl 118, KCl 5.4, NaHCO_3_ 24, MgCl_2_1.02, CaCl_2_ 1.8, NaH_2_PO_4_ 0.42, dextrose 12, and taurine 20, bubbled with 95% O_2_ and 5% CO_2_, pH 7.4, at 37°C, followed by perfusion with the same solution without Ca^2+^ to which collagenase type II (0.5 mg/mL) and protease type XIV (0.03 mg/mL) were added later. Hearts were finally perfused with KB solution (in mmol/L): taurine 10, glutamic acid 70, creatine 0.5, succinic acid 5, dextrose 10, KH_2_PO_4_ 10, KCl 20, HEPES^-^K^+^ 10, EGTA^-^K^+^ 0.2, pH adjusted to 7.4 with KOH. Then, the ventricular cardiomyocytes were dissociated by mechanical agitation. When isolated for cardiac calcium handling experiments, EGTA was not included in the KB solution.

### In vitro *electrophysiology patch-clamp experiments*

All experiments were performed at room temperature (20–22°C). Following the patch rupture, the whole-cell membrane capacitance was measured by integration of the capacitive transients elicited by voltage steps from −50 to −60 mV. Series resistance was compensated 80% in order to minimize voltage errors.

Ionic currents were recorded using the whole-cell configuration with an Axopatch 200B patch-clamp amplifier (Molecular Devices). Recording pipettes pulled from borosilicate tubes (Sutter Instruments) had a tip resistance of 1–3 MΩ when filled with the internal solution. The pipette solution used for I_to_ and I_Ca-L_ recording was (in mmol/L): L-aspartic acid (potassium salt) 80, KH_2_PO_4_ 10, MgSO_4_ 1, KCl 50, HEPES^-^K^+^ 5, ATP^-^Na_2_ 3, EGTA^-^K^+^ 10, pH adjusted to 7.2 with KOH. For the current carried by NCX (I_NCX_), the internal solution was: CsCl 115, NaCl 10, TEA-Cl 10, Mg-ATP 5, MgCl_2_ 0.5, HEPES 10, pH adjusted to 7.2 with CsOH.

For I_to_ recording, the bath solution was (in mmol/L): NaCl 86, MgCl_2_ 1, HEPES^-^Na^+^ 10, KCl 4, CaCl_2_ 0.5, CoCl_2_ 2, dextrose 11, TEA-Cl 50, pH adjusted to 7.4 with NaOH. For I_Ca_-L, the solution composition was (in mmol/L): NaCl 86, MgCl_2_ 1, HEPES^-^Na^+^ 10, KCl 4, CaCl_2_1.8, TEA-Cl 50, 4-aminoprirydine 4, dextrose 11, pH adjusted to 7.4 with NaOH. Voltage pulses (500 ms long) to a test potential ranging from −30 to +50 mV, starting from a holding potential of −60 mV, were applied at a frequency of 0.1 Hz to ensure the full channel recovery from inactivation, whereas for I_Ca_-L recording, the 500 ms test pulses were preceded by a 40 ms pre-pulse to −40 mV.

For recording I_NCX_, the external solution was (in mmol/L): CsCl 10, NaCl 130, MgCl_2_ 0.5, CaCl_2_ 1, HEPES 10, dextrose 10, pH adjusted to 7.4 with NaOH. The current was recorded during application of a ramp from +80 to −140 mV following a 150 ms step to +80 mV, from a holding potential of −90 mV, after sequential pre-pulses to −45 and 0 mV to inactivate Na^+^ and Ca^2+^ channels, respectively. The protocol was repeated in the presence of 10 mM NiCl_2_ to inhibit NCX. I_NCX_ was considered as the difference of the currents recorded in the absence and presence of Ni^2+^.

The voltage-clamp experimental protocols were controlled with the “Clampex” program, and current recordings were analyzed with the “Clampfit” program of the pClamp10.2 software (Molecular Devices). Current amplitudes were normalized to cell capacitance and expressed as pA/pF.

### Measurement of [Ca^2+^]_i_

After 24 hours of incubation at 4°C with EGTA-free KB solution with or without 30 mIU/L TSH, [Ca^2+^]_i_ was measured in rat myocytes loaded with indo-1 AM (Molecular Probes) for 15 minutes followed by 30 minutes of washout. Cells were perfused at 22–24°C with Tyrode's solution containing 1 mmol/L CaCl_2_. The fluorescence emission ratio at 410 and 485 nm under 360 nm excitation was converted to [Ca^2+^]_I_, as described elsewhere (11). Ca^2+^ transients were recorded after interruption of 1 Hz electrical stimulation followed by switching perfusion to normal or Na^+^, Ca^2+^-free Tyrode solution containing 10 mmol/L caffeine. The rate constant of [Ca^2+^]_i_ decline (K) was determined by nonlinear curve fit. Sustained activation of sarcoplasmic reticulum (SR) Ca^2+^ release channels by caffeine prevents net Ca^2+^ accumulation in the organelle, so that most of [Ca^2+^]_i_ decline should be carried by the NCX, if the external solution contains Na^+^, whereas the exchanger is thermodynamically inhibited in Na^+^-free medium. Thus, the ratio of K determined in the presence and absence of extracellular Na^+^ can be used as an index of the NCX activity in the Ca^2+^ efflux mode (12).

The effect of β-adrenergic stimulation at inducing spontaneous activity was assessed by determination of concentration–effect curves to isoproterenol (ISO), in which the rate of spontaneous Ca^2+^ transients was measured after interruption of electrical stimulation.

### Quantitative real-time polymerase chain reaction

This study conforms to the principles outlined in the Declaration of Helsinki and was approved by the Ethics Committee of the Instituto Nacional de Cardiologia (CEP-0358/07-11-2011). Human right atrial samples were obtained from patients in sinus rhythm who underwent cardiac surgery (*n* = 8). Clinical data of the patients are included in Supplementary Table S1.

Just after excision, each sample was cut in pieces that were incubated for 24 hours at 4°C in the absence or in the presence of TSH, either with or without of 5 μmol/L of the PKA blocker H89, after which the levels of mRNA expression of the *KCND3*, *KCNQ1*, and *KCNH2* genes were measured by quantitative reverse transcription polymerase chain reaction. Total RNA was extracted from 10 mg of frozen tissue treated with DNase using the RNeasy^®^ Fibrous Tissue Mini Kit (Qiagen) following the manufacturer's instructions. The concentration of total RNA in each sample was measured spectrophotometrically using NanoDrop 2000 (Thermo Fisher Scientific). Next, 1 μg of total RNA was reversely transcribed into cDNA using random primers and the High Capacity Reverse Transcription Kit (Applied Biosystems). The reactions were carried out in the PTC-100 Programmable Thermal Controller (MJ Research). The program was 25°C for 10 minutes, 37°C for 120 minutes, 85°C for 5 minutes, and 4°C for 10 minutes.

Determinations were performed on 96-well plates in 15 μL final volume containing 2 μL of 10 × diluted *KCND3*, *KCNH2*, and *KCNQ1* cDNA stock, 7.5 μL of Power SYBR Master Mix (Applied Biosystems), and 150 mmol/L of each forward and reverse of *KCND3*, *KCNH2*, and *KCNQ1* primers (see Supplementary Table S2). The amplification program was 55°C for 2 minutes, 95°C for 10 minutes, followed by 40 cycles of 95°C for 30 seconds and 60°C for 1 minute. Each cDNA in duplicate and a corresponding sample without reverse transcriptase were included as a negative control. The mRNA expression of the chosen genes was normalized to that of *GAPDH* used as an internal control. The relative quantities of gene-specific mRNA were determined by the comparative Ct method, by which the relative gene expression in the TSH-treated group is expressed by the formula 2^(ΔΔCt)^, where Ct refers to the “threshold cycle” determined for each plate by the ViiA™ 7 Real-Time PCR System (Applied Biosystems); ΔCt is the difference between the Ct values of the target mRNA and that of GAPDH; and ΔΔCt is the difference between the mean ΔCt of the TSH and control groups. The fold-change in the expression of the target genes was calculated as the mean ± standard deviation of 2^(ΔΔCt)^ for the control and TSH groups.

### Computational modeling

Human ventricular action potentials (AP) of 550 control individuals in resting conditions and under β-adrenergic stimulation were simulated using the O'Hara–Rudy dynamic (ORd) model (13) as baseline. Following the methodology by Passini *et al*. (14), ionic conductances were set at 20–200% of the baseline values of the ORd “average human” to include ion current profiles with variable channel densities while keeping the AP parameters (peak voltage, maximum upstroke velocity, resting membrane potential, AP duration at 50% and 90% repolarization, and triangulation) within the normal range. The main ionic conductances considered were: fast and late Na^+^ currents (G_Na_ and G_NaL_, respectively), I_to_ (G_to_), I_Kr_ and I_Ks_ (G_Kr_ and G_Ks_), inward rectified K^+^ current (G_K1_), I_NCX_ (G_NCX_), Na^+^/K^+^ pump (G_NaK_), and I_Ca-L_ (G_Ca-L_). All simulations presented in this study were conducted using Virtual Assay (© 2016, University of Oxford; v.1.3.640 2014; Oxford University Innovation Ltd.), a software package for *in silico* drug assays, kindly provided by B. Rodríguez.

In order to reproduce the hypothyroid phenotype in this population of 550 AP models, ionic conductances were modified according to the experimental findings in both a past study (10) and the present study. To simulate the change in I_Ca-L_ in both types of hypothyroidism, G_Ca-L_ was augmented by 55%. To simulate the changes associated with primary hypothyroidism, G_to_ and G_K1_ were reduced to 50% of control, whereas G_Ks_ and G_NCX_ were reduced to 60% and 70% of control, respectively. In the simulations, trains of 500 AP were evoked at 1 Hz, and the last AP trace in each simulation was presented.

Simulations of the impact of the ion current changes observed in hypothyroidism were also performed on the Nygren–Firek–Clark–Lindblad–Clark–Giles model (15) of human atrial AP. The simulations presented in this study were conducted with permission using The Virtual Heart Web page by Flavio Fenton (16–18), and are described in the Supplementary Methods.

### Statistical analysis

Data are presented as means ± standard error of the mean. Data were compared with Student's *t*-test or analysis of variance followed by Bonferroni's *t*-test. Arrhythmia incidence was evaluated with a contingency table and Pearson's chi-square test. A *p*-value of <0.05 was considered as statistically significant.

## Results

### Animal models

Although PTU treatment is a widely used procedure for induction of primary hypothyroidism, to the best of the authors' knowledge, an animal model of central hypothyroidism had not yet been developed. The anticancer drug BXT exerts a direct inhibitory effect on the TSH-producing cells of the pituitary gland (19,20). Advantage was taken of this unwanted side effect to create a central hypothyroidism experimental model, which was tested in this study.

**FIG. 1.**
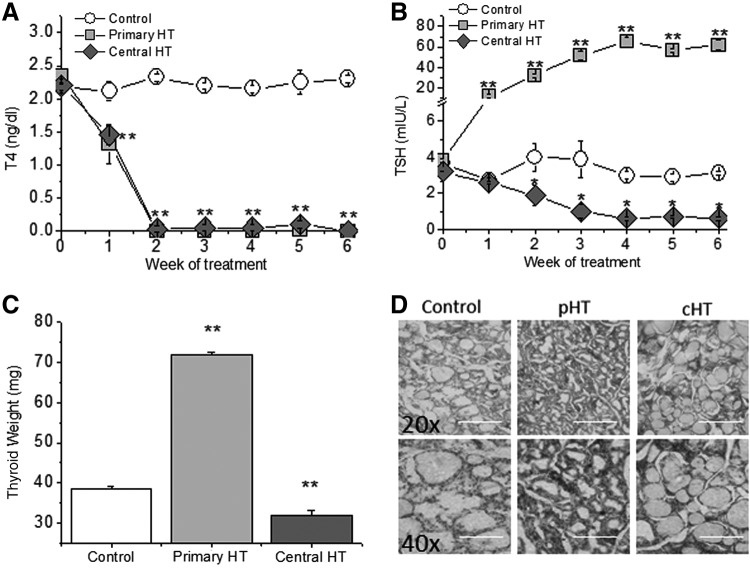
Characterization of the hypothyroidism animal models. (**A**) Free thyroxine (T4) blood levels are almost undetectable after two weeks of either primary or central hypothyroidism. (**B**) Serum thyrotropin (TSH) levels were increased more than 10-fold in primary hypothyroidism but were undetectable in animals with central hypothyroidism. (**C**) Thyroid gland weight showed opposite changes in primary and central hypothyroidism after six weeks of induction. (**D**) Hematoxylin and eosin stained sections of the thyroid gland six weeks after induction of primary (pHT) and central hypothyroidism (cHT) also show opposite structural modifications. Scale bars indicate 200  μm and 100 × μm at 20 × and 40 × , respectively. *n* = 18 control, 15 pHT, and 17 cHT animals. Means ± standard error of the mean (SEM). *p < 0.05; **p < 0.01.

Both PTU and BXT treatments reduced blood T4 concentration within one week, attaining almost undetectable levels after two weeks (Fig. 1A). Since PTU directly induces malfunction of the thyroid gland, blood TSH concentration showed a 10-fold increase, reaching its maximum at week 4. BXT, on the other hand, is expected to reduce T4 levels due to its toxic effect on the pituitary gland. Accordingly, TSH levels were reduced, reaching the minimum at week 4 (Fig. 1B).

After six weeks of primary hypothyroidism, the high circulating TSH levels induced thyroid growth with an increase in epithelial cell height, whereas in low TSH central hypothyroidism, gland size and follicular cells height were slightly reduced (Fig. 1C and D and Supplementary Fig. 1A). Primary hypothyroidism reduced body weight, which was not changed in control or centrally hypothyroid animals. Neither body temperature nor relative heart mass was significantly different among the three groups of animals (Supplementary Fig. 1B–D).

### Electrocardiographic characteristics

**FIG. 2.**
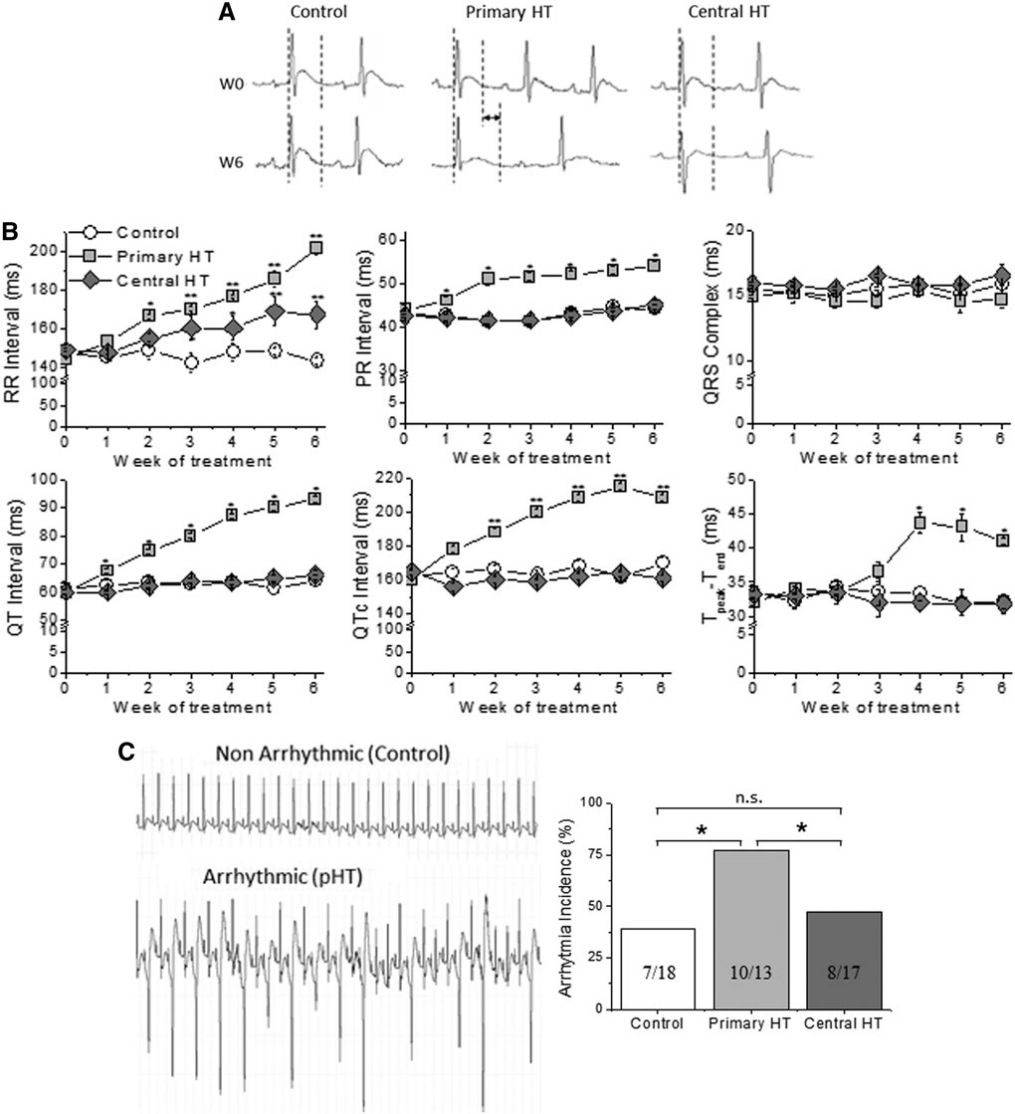
Electrocardiographic (ECG) characteristics of central and primary hypothyroidism. (**A**) ECG recordings before (W0) and after six weeks of hypothyroidism (W6). The dotted lines delineate the QT interval. (**B**) Both central and primary HT prolonged the R-R interval. Only primary HT prolonged the QTc, PR and QT intervals, and Tpeak-to-Tend duration. QRS complex duration was not modified in any HT model (*n* = 18, 13, and 17 animals/group, respectively). (**C**) After caffeine/dobutamine challenge, the incidence of ventricular arrhythmias was increased twofold only in the primary hypothyroid group compared to control animals. The numbers in the columns are arrhythmic animals/total animals in the group. Data were compared with analysis of variance followed by Bonferroni's *t*-test. Means ± SEM. *p < 0.05; **p < 0.01.

As previously published (21), primary hypothyroidism reduced heart rate compared to control animals, whereas a less pronounced effect was seen in the centrally hypothyroid group (Fig. 2A and B). Similarly to patients with primary hypothyroidism (5), the primary hypothyroidism group showed repolarization abnormalities, such as prolongation of the QT, QTc, and Tpeak-to-Tend intervals. However, none of these alterations was present in centrally hypothyroid animals (Fig. 2B).

The ECG alterations seen in hypothyroid animals may represent a substrate for the development of cardiac arrhythmias. Thus, the animals were subjected to an arrhythmia vulnerability test under acute challenge with caffeine and dobutamine. The incidence of cardiac arrhythmias was increased twofold in animals with primary hypothyroidism, while only a nonsignificant trend of increase was seen in the centrally hypothyroid group (Fig. 2C).

### Cardiac electrical remodeling

**FIG. 3.**
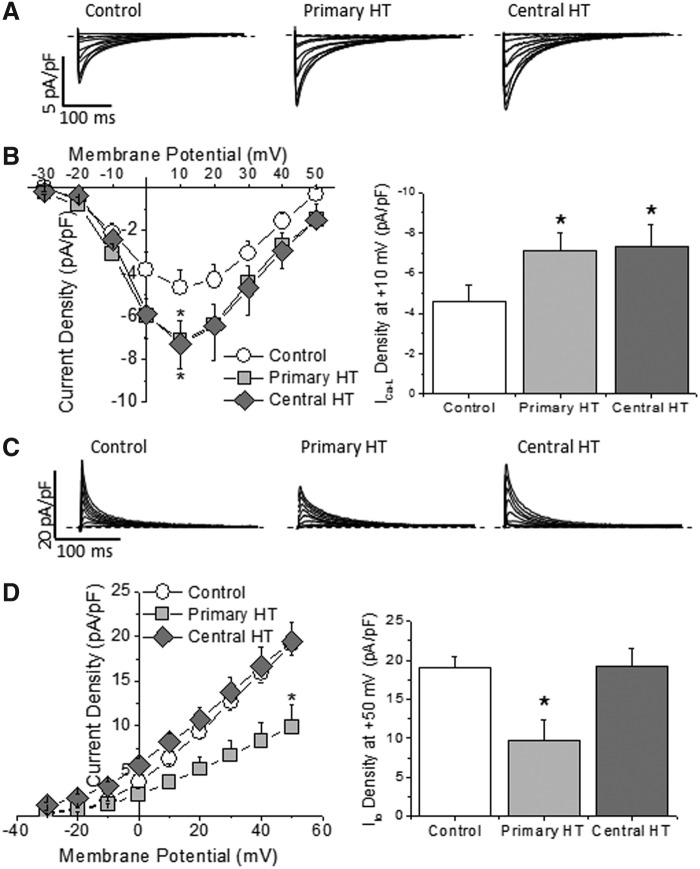
Hypothyroidism differently modulated Ca^2+^ and K^+^ currents. (**A**) I_Ca-L_ traces recorded in ventricular myocytes isolated from control animals and from animals with primary and central hypothyroidism. Dashed lines represent the 0 current level. (**B**) Current–voltage relationship and maximum I_Ca-L_ amplitude at +10 mV showing a similar increase in I_Ca-L_ density in both central and primary hypothyroidism (*n* = 8–15 cells from at least three animals in each group; *p < 0.05). (**C**) Traces of I_to_ recorded in ventricular myocytes isolated from control animals and from animals with primary and central hypothyroidism. Dashed lines represent the 0 current level. (**D**) Current–voltage relationships and maximum I_to_ amplitude at +50 mV, showing that primary hypothyroidism reduced I_to_ density, whereas hypothyroidism of central origin had no effect on this current (*n* = 10–15 cells from at least three animals in each group; *p < 0.05).

The amplitude of I_Ca-L_ in ventricular myocytes increased in both primary and central hypothyroidism when compared to the control group (Fig. 3A and B). These results support the reported absence of effect of TSH on this current (10) and the suppressive effect of thyroid hormones on L-type Ca^2+^ channels, since T3/T4 deficiency increases both calcium channel expression and current amplitude (22,23).

In ventricular myocytes isolated from animals with primary hypothyroidism, the I_to_ amplitude was reduced, but it was not affected by central hypothyroidism (Fig. 3C and D). In addition, hypothyroidism modified neither the voltage-dependence of the steady-state inactivation of the current nor the time-dependence of recovery from inactivation (Supplementary Fig. 2A and B). Thus, although primary hypothyroidism reduced I_to_ density, the biophysical properties of the channel were not modified, suggesting a reduction of the channel abundance. Accordingly, expression of the Kv4.2 and Kv4.3 channel proteins was significantly reduced in ventricles from rats with primary hypothyroidism, but it was conserved in hypothyroidism of central origin (Supplementary Fig. 2C). Thus, the absence of thyroid hormones does not seem to be the mechanism responsible for the reduction in I_to_ density and channel expression in rats with primary hypothyroidism, pointing to high TSH levels as the likely factor involved in these changes.

Although its physiological relevance is lower than I_to_, the ultra-rapid delayed rectifier K^+^ current I_Kur_ is also present in the rat ventricle. In ventricular myocytes, the I_Kur_ amplitude was similarly decreased in both primary and central hypothyroidism when compared to the control group (Supplementary Fig. 3A and B). As for I_Ca-L_, these results support a direct role of thyroid hormones and lack of influence of TSH on this current, as previously reported (10).

### Role of TSH in electrical remodeling

**FIG. 4.**
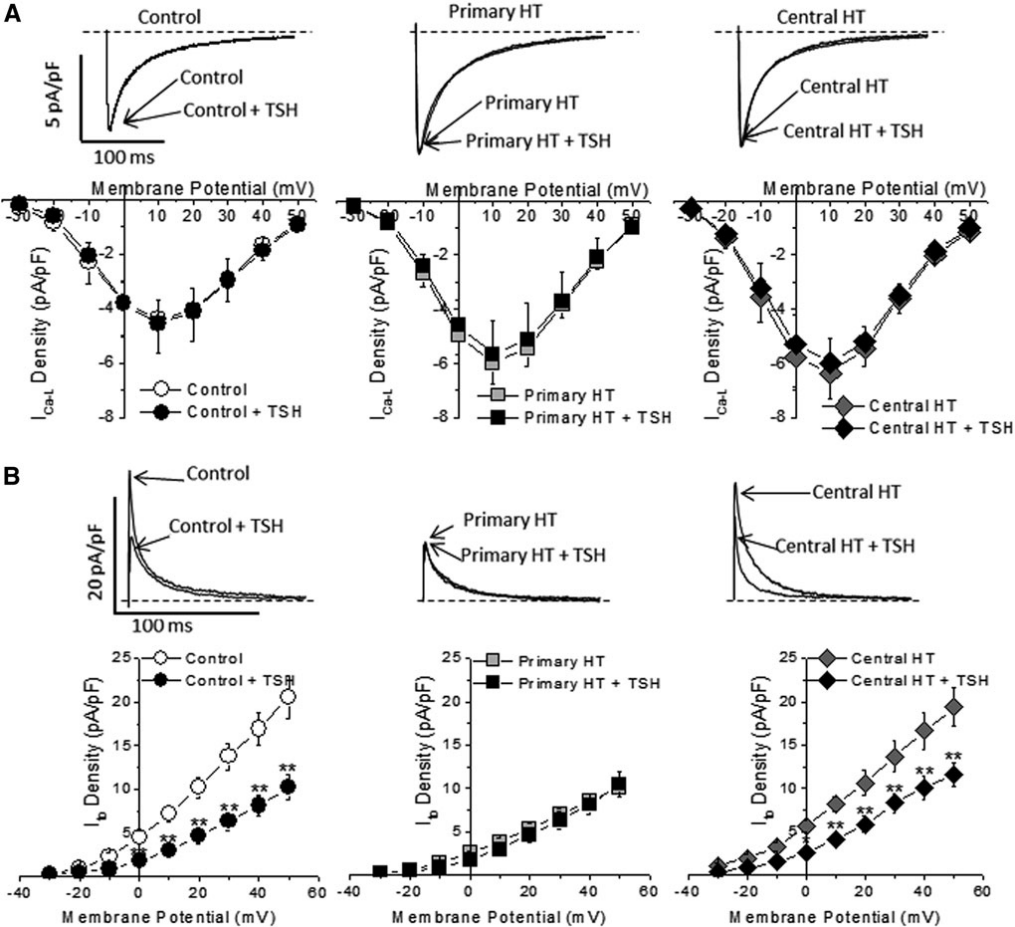
TSH modulation of Ca^2+^ and K^+^ currents in myocytes from control animals and rats with either central or primary hypothyroidism. (**A**) Incubation for 24 hours with 30 mIU/L of TSH did not affect I_Ca-L_ in any of the experimental groups (*n* = 8–9 cells from three different animals in each group). (**B**) Incubation with TSH reduced the I_to_ amplitude in the control and central hypothyroid groups, but had no effect in the primary hypothyroidism group. Means ± SEM. *n* = 10–13 cells from at least three different animals in each group. Dashed lines represent the 0 current level. *p < 0.05; **p < 0.01.

Next, the study explored at the cellular level a possible direct role of TSH in the electrophysiological changes seen in animal models of hypothyroidism. Isolated ventricular myocytes were incubated for 24 hours in medium containing 30 mIU/L of TSH, a concentration commonly seen in primary hypothyroid patients. As shown in Figure 4A, TSH had no effect on I_Ca-L_ either in control or in the hypothyroid groups.

On the other hand, incubation with TSH differentially modulated the I_to_ amplitude in the three experimental groups. While exposure to TSH significantly reduced the I_to_ amplitude in myocytes from control and centrally hypothyroid rats (Fig. 4B), exogenous TSH had no effect on I_to_ density in myocytes from animals with primary hypothyroidism, a model with high circulating TSH levels in which I_to_ was already decreased (Fig. 4B).

### TSH effect on cardiomyocyte Ca^2+^ handling

**FIG. 5.**
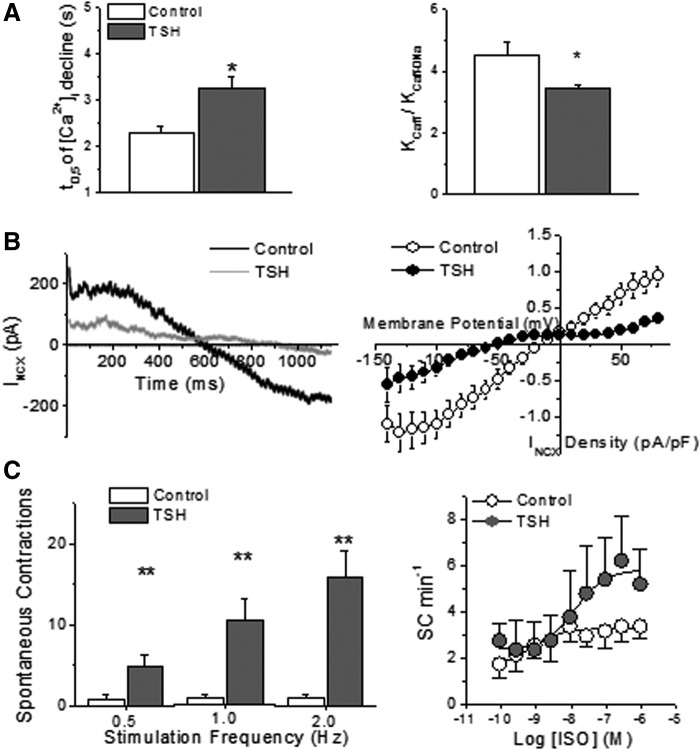
Effects of *in vitro* TSH treatment on cardiomyocyte Ca^2+^ handling. (**A**) TSH increased the half-time (t_0.5_) of [Ca^2+^]_i_ decay of caffeine-evoked transients, which depends mainly on NCX, and decreased the ratio of the rate constants of [Ca^2+^]_i_ decline of caffeine transients in the presence (K_caff_) and absence of Na^+^ (K_caff-0Na_; *n* = 27 in control and 29 in the TSH group). These results indicate a reduction of NCX activity in intact myocytes. (**B**) Recordings of the I_NCX_ in isolated ventricular myocytes confirm the reduction of NCX activity by TSH (*n* = 8 and 10 cells for control and TSH groups, respectively). (**C**) TSH increased the number of spontaneous contractions (SC) during 200 seconds after a 10-second stimulation train at different frequencies (*n* = 18, 22, 16, 18, 11, and 9 for each column, respectively). TSH treatment also enhanced the concentration-dependent induction of automatism by isoproterenol (ISO) in rat ventricular myocytes developed after interruption of electrical stimulation. *n* = 5 cells in each group. Means ± SEM. *p < 0.05.

NCX has been implicated in the development of Ca^2+^-dependent spontaneous electrical activity in myocardial cells (24,25). Incubation of control myocytes with TSH for 24 hours did not cause apparent cellular Ca^2+^ overload. However, TSH exposure significantly prolonged the decay phase of Ca^2+^ transients generated by caffeine-induced massive SR Ca^2+^ release, which relies mainly on NCX (Fig. 5A, left). The decrease in the ratio of the time constants for decline of the cytosolic Ca^2+^ concentration of caffeine-evoked transients in the presence and absence of Na^+^ after TSH incubation (Fig. 5A, right) confirmed that the hormone depressed the Na^+^-dependent Ca^2+^ efflux. Accordingly, the I_NCX_ amplitude was smaller (*p* < 0.001) in cells preincubated with TSH, over the whole tested voltage range (Fig. 5B). Nevertheless, TSH treatment caused a leftward shift in the current reversal potential from −17.4 ± 4.9 to −46.5 ± 6.4 mV (*p* < 0.01).

Finally, the effect of TSH incubation on the development of spontaneous contractions after interruption of electrical stimulation was tested in the absence and presence of the β-adrenoceptor agonist ISO. In the absence of the agonist, TSH-treated cells showed greater incidence of spontaneous activity after a brief (10 seconds) train of stimulation at all tested rates, from 0.5 to 2 Hz (Fig. 5C, left). Another stimulation protocol (1 Hz for 2 minutes) (26) was used to test β-adrenergic stimulation with ISO. Although ISO was effective at inducing automatism in cells incubated with and without TSH (*p* < 0.001), the response was more prominent in the latter group (*p* < 0.05 for treatment × [ISO] interaction), in which the maximum rate of spontaneous events was twofold greater (Fig. 5C, right).

### Effects of TSH on human cardiac myocytes

**FIG. 6.**
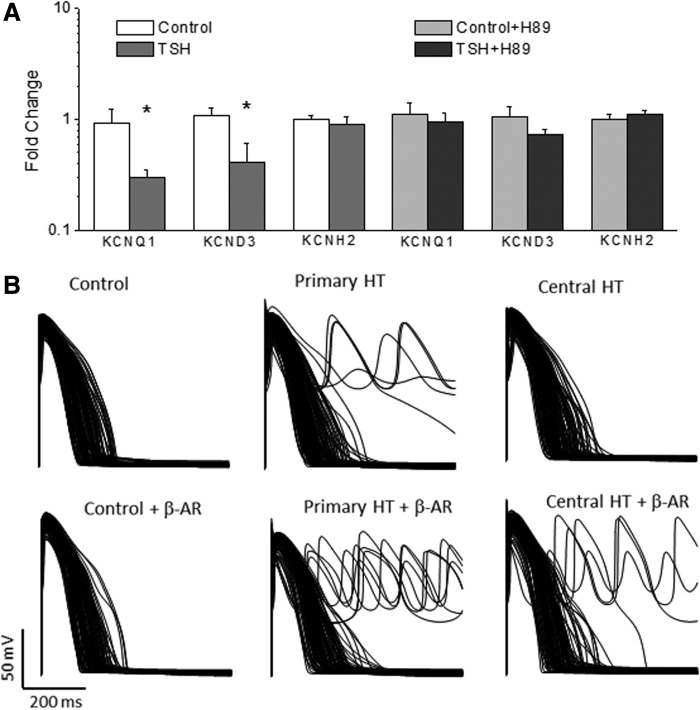
Effects of TSH on human cardiac myocytes *in vitro* and *in silico.* (**A**) mRNA levels of *KCNQ1* and *KCND3* were reduced in TSH-treated adult human atrial myocytes when compared to control myocytes, whereas *KCNH2* expression was not affected. The effect of TSH was fully abolished by 5 μM of the PKA blocker H89. *n* = 8 hearts. Means ± SEM. *p < 0.05. (**B**) Computational models were generated to simulate the action potential of 550 control individuals in resting conditions (upper panel) and under β-adrenergic stimulation (lower panel). Only 110 randomly selected traces are shown for clarity. When the ionic conductances were changed to simulate the effects of primary hypothyroidism, early afterdepolarization (EAD) and arrhythmia appeared in resting conditions and increased after beta-adrenergic stimulation. When ionic conductances were adjusted to simulate central hypothyroidism, EAD and arrhythmia were absent in resting conditions but present after β-adrenergic stimulation.

Next, the study explored whether the effects of TSH on cardiac electrical properties observed in rodents also occurred in human cardiomyocytes. Thus, the study tested whether TSH incubation for 24 hours affects the expression of the channels that carry repolarizing K^+^ currents in adult human myocytes. TSH reduced the expression of the *KCND3* and *KCNQ1* genes, which might result in a reduction of I_to_ and I_Ks_, as seen in rodents in both prior studies (10,22) and in the present study. TSH, however, did not affect the expression of *KCNH2*, responsible for the I_Kr_ current in human heart (Fig. 6A).

Next, the study explored the molecular mechanism involved in the effect of TSH on K^+^ channel expression in the heart. The TSH receptor is canonically coupled to a Gsα protein and activates the adenylyl cyclase/PKA pathway. To test the effect of PKA inhibition, samples were treated with TSH for 24 hours in the presence of H89, which fully eliminated the effect of TSH on the expression of the studied channels (Fig. 6A).

### Computer simulation of human AP

A population of 550 human ventricular AP profiles was simulated with different ion channel densities, by sampling the main ventricular ionic conductances in the 20–200% range of the averaged values in the ORd model (13), either under resting conditions or after β-adrenergic stimulation (Fig. 6B). When ionic conductances were changed to simulate the alterations seen in the primary hypothyroid conditions (see Methods), 24/550 models showed EAD and arrhythmias under resting conditions, and the incidence increased to 33/550 after β-adrenergic stimulation. When ionic conductances were adjusted to simulate central hypothyroidism, EAD was not observed under resting conditions, but after β-adrenergic stimulation, EADs and arrhythmias were generated in 26/550 cases.

The same kind of simulation carried out with the Nygren–Firek–Clark–Lindblad–Clark–Giles model (15) of atrial AP reproduced the results obtained with the ventricular model: arrhythmias appeared in the primary hypothyroidism model, whereas only isolated EADs were seen in the central hypothyroidism model (Supplementary Fig. S4).

## Discussion

For decades, PTU-induced hypothyroidism has been the most widely used animal model (21,27). After PTU treatment, T4 becomes undetectable, and TSH increases more than 10-fold. However, to the best of the authors' knowledge, a well-characterized animal model of central hypothyroidism has been lacking. The rexinoid drug BXT (28,29) induces reversible central hypothyroidism in both patients and experimental animals (19,20). In the present work, both T4 and TSH levels were very significantly reduced after BXT treatment, resulting in a sustained hypothyroid status. In conclusion, BXT treatment (50 mg/kg by daily gavage) generates a new and reliable animal model of central hypothyroidism.

It is well known that secondary hypothyroidism does not produce the same clinical and biochemical degree of hypothyroidism as primary thyroid underfunction. In addition, secondary hypothyroidism is usually a part of a more complex phenotype, with anterior pituitary insufficiency or pan-hypopituitarism caused by entities such as pituitary/hypothalamic neoplasia or infiltration, surgery, or irradiation (3,30). However, the severity of hypothyroidism found in the PTU and BXT animal models used in this study is similar, since T4 is undetectable in both. Many (probably most) cardiac and systemic manifestations of primary hypothyroidism are directly related to the lack of thyroid hormones. However, the novelty of the study presented here is to present data that demonstrate that a subset of the cardiac electrical remodeling is associated with the high TSH levels found in primary hypothyroidism.

The prolongation of the QTc interval seen in ECGs of hypothyroid patients was reproduced in animals with primary hypothyroidism but not in the central hypothyroid model, which suggests that it is not solely dependent on thyroid hormone deficiency. In addition, the incidence of arrhythmias in rats with primary hypothyroidism was twofold elevated but not observed in animals with central hypothyroidism. Thus, we conclude that hypothyroidism-induced increase of the ventricular vulnerability to arrhythmia is associated with the high serum TSH levels.

Thyroid hormones inhibit the expression of L-type Ca^2+^ channels (23), and therefore, in primary hypothyroidism, *CaCN1C* gene expression increases (22). Accordingly, the present results show an increase of the I_Ca-L_ amplitude in the two models of hypothyroidism, supporting the causative role of the decreased levels of thyroid hormones. On the other hand, it was also observed that I_Kur_ was reduced in both hypothyroid models, as in previous studies (22,31). This indicates that both the I_Ca,L_ and the I_Kur_ current are regulated by thyroid hormones but not by TSH, as previously reported (10). However, although the increase in I_Ca-L_ and the reduction in the I_Kur_ amplitude should contribute to prolonged repolarization, this appears to occur only in primary but not in central hypothyroidism, suggesting that there must be other mechanisms involved in the ventricular electrical remodeling. Nevertheless, these changes might, at least in part, explain the incidence of atrial arrhythmias in patients with hypothyroidism.

A classical trigger of arrhythmias is the depolarizing current carried by NCX, secondary to cellular Ca^2+^ overload (24–26). However, the current results indicate that this is not the trigger mechanism of the TSH-induced arrhythmogenesis. It was found that 24 hours of incubation of isolated cardiac myocytes with TSH did not cause Ca^2+^ overload and reduced the pro-arrhythmic I_NCX_ current. Conversely, during β-adrenergic stimulation, the diastolic Ca^2+^ leak from the sarcoplasmic reticulum is enhanced due to greater Ca^2+^ accumulation in the organelle (with contribution of I_Ca-L_ stimulation, already augmented by hypothyroidism) and by indirect stimulation of Ca^2+^ releasing channels (24,32). A greater Ca^2+^ leak is expected to enhance I_NCX_ markedly because it increases the availability of subsarcolemmal Ca^2+^ to be transported by the NCX. This, added to the prolonged repolarization observed only after exposure to supranormal TSH levels (TSH incubation and primary hypothyroidism), is expected to promote pro-arrhythmic afterdepolarizations. It should be noted that in animals with both primary hypothyroidism and TSH-treated isolated myocytes, enhanced spontaneous/arrhythmic activity compared to controls was seen under β-adrenergic stimulation. Beta-adrenergic stimulation was also found to result in a marked increase in ventricular pro-arrhythmic events in primary hypothyroidism. Overall, these findings indicate that that the electrical remodeling induced by TSH overexposure may sensitize the myocardium to the pro-arrhythmic influence of adrenergic stimulation, such as during exercise and under stressful conditions.

Although thyroid hormones are necessary for the I_to_ development in neonatal cardiac myocytes (33–35), it was consistently observed that thyroid hormones have no effect on I_to_ in cardiac myocytes from either euthyroid or hypothyroid adult animals (35,36). Thus, while the increase in I_Ca-L_ in hypothyroid animals can be explained by the decrease in thyroid hormones, this does not apply to the decrease of I_to_. The present results suggest a direct action of TSH as the mechanism responsible for the I_to_ reduction. In accordance with this hypothesis and with previously published data (10,21,22), ventricular I_to_ was reduced in the high TSH model of primary hypothyroidism but not in the low TSH central hypothyroid model. In addition, the *in vitro* suppressive effect of TSH on I_to_ seen in myocytes from controls could not be reproduced in cardiomyocytes isolated from rats with primary hypothyroidism, in whom serum TSH levels were elevated for six weeks and I_to_ was already decreased. In contrast, incubation with TSH was able to reduce I_to_ in cardiomyocytes from rats with central hypothyroidism, in whom TSH levels were not elevated and the I_to_ amplitude was not altered. All these results indicate a central role of TSH in the cellular electrical remodeling in primary hypothyroidism.

Although the prolongation of repolarization time seen in rodents is mainly associated with I_to_ inhibition, in humans, alterations of other K^+^ currents cannot be excluded. Thus, the possible effects of TSH on human atrial samples were explored. TSH reduced the expression of the *KCND3* and *KCNQ1* genes, which encode the channels that mediate I_to_ and I_Ks_ respectively. This effect is likely to contribute to the prolonged repolarization reported in patients with primary hypothyroidism.

The presence of TSH receptors in the heart was previously reported both at the mRNA and protein levels, and these receptors appear to be functional and coupled to the adenylyl cyclase-PKA pathway (37,38). The current and previously reported results (10) demonstrate that either TSH receptor blockade or PKA inhibition abolishes the effect of the hormone on cardiac K^+^ channel expression, indicating that TSH acts through its canonical pathway in the heart.

To translate to the human heart the experimental data on susceptibility to triggered arrhythmia in hypothyroidism, computer simulations of the electrophysiological behavior of human cardiomyocytes were used. When the primary hypothyroid phenotype was introduced in the models, EADs and arrhythmias appeared, and their incidence increased after β-adrenergic stimulation, consistent with the higher incidence of arrhythmias in patients with primary hypothyroidism (5) and with the current *in vivo* and *in vitro* experimental results. It is also interesting to note that EADs and arrhythmias emerged only under β-adrenergic stimulation in the central hypothyroidism models. As pointed out, an increase in I_Ca-L_ reportedly due to thyroid hormone deficiency may contribute to EAD triggering during β-adrenergic stimulation in individuals with either type of hypothyroidism.

From a clinical perspective, several independent studies proposed that elevated serum TSH levels play an essential causal role in the QT prolongation and dispersion (QTd) in patients with overt hypothyroidism. These abnormalities revert after hormone replacement and normalization of TSH levels (4,5,8). Here, based on the observations of the direct effect of TSH on the K^+^ currents, a mechanistic explanation is proposed for the TSH-dependent electrocardiographic alterations and its possible contribution to enhanced susceptibility to cardiac arrhythmias. These results, together with the recently published association between high normal TSH levels and increased risk of mortality, further contribute to answering the question of whether serum TSH reference ranges should be reexamined (1,2,39,40).

## Supplementary Material

Supplemental data
