# Supplementary material for: High Thyrotropin Is Critical for Cardiac Electrical Remodeling and Arrhythmia Vulnerability in Hypothyroidism
Source: Thyroid. 2019 Jul 17;29(7):934–45. doi: 10.1089/thy.2018.0709 (PMC6648210; doi:10.1089/thy.2018.0709)
Supplement: Supplemental data [file Supp_Data.pdf]

Supplementary Methods

Western Blot

Total lysate of isolated rat cardiomyocytes (50 mg) in RIPA buffer were fractionated on 8% sodium dodecyl sulfate polyacrylamide gels and transferred to nitrocellulose membranes (Amersham Biosciences). Ponceau staining was used for protein loading control. Nitrocellulose membranes were then blocked in TTBS solution (Tris-HCl 50 mmol/L, NaCl 150 mmol/L, Tween-20 0.05%; pH 7.5) containing 5% nonfat dry milk (BioRad). Blots were incubated with goat antibodies anti-Kv4.2 (1:200) and anti-Kv4.3 (1:200) from Santa Cruz Biotechnology, and then with secondary donkey anti-goat antibody (1:5000; Santa Cruz Biotechnology). Blots were developed using chemiluminescence (West pico; Pierce) and images were acquired with a GelLogic 2200 image station (Kodak). The bands were analyzed with the Kodak Molecular Imaging 4.0.4 software (Eastman Kodak Company).

Computational Modeling of Atrial Action Potential

Human atrial action potential of a control individual in resting conditions was simulated using the Nygren–Firek–Clark–Lindblad–Clark–Giles model (S1). The simulations presented in this study were conducted with permission using The Virtual Heart page by Flavio Fenton (S2). In order to reproduce the hypothyroid phenotype ionic conductances were modified according to previous experimental findings (S3) and the present results. To simulate the change in $I_{Ca\text{-}L}$ and I_{Kur} in both types of hypothyroidism, they were augmented by 55% and reduced by 27%, respectively. To simulate the changes associated with primary hypothyroidism, G_{to} and G_{K1} were reduced to 50% of control, whereas G_{Ks} and G_{NCX} were reduced to 60% and 70% of control, respectively. In the simulations, trains of 10 AP were evoked at 1 Hz, and the complete train was presented.

Supplementary References

S1. Nygren A, Fiset C, Firek L, Clark JW, Lindblad DS, Clark RB, Giles WR 1998 Mathematical model of an adult human atrial cell: the role of K^+ currents in repolarization. Circ Res **82:**63–81.

S2. Fenton F, Cherry E. The Virtual Heart. Available at: http://dev1.thevirtualheart.org/ (accessed January 16, 2019).

S3. Alonso H, Fernández-Ruocco J, Gallego M, Malagueta-Vieira LL, Rodríguez-de-Yurre A, Medei E, Casis O 2015 Thyroid stimulating hormone directly modulates cardiac electrical activity. J Mol Cel Cardiol **89:**280–286.

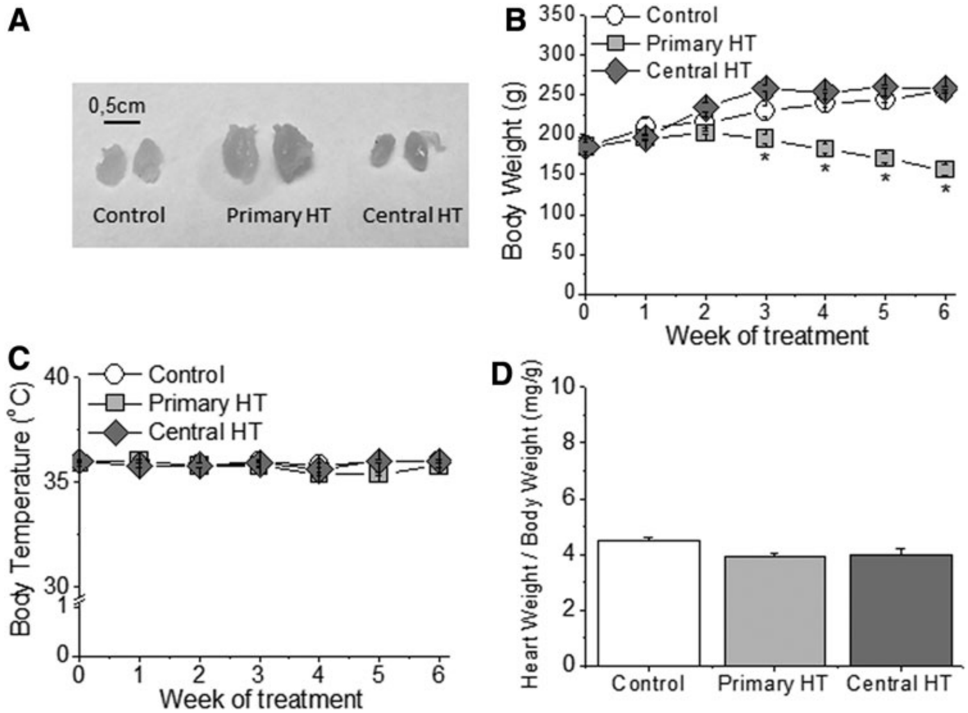

SUPPLEMENTARY FIG. S1. Characterization of hypothyroidism animal models. (**A**) As expected, only the thyroid glands of rats with primary hypothyroidism showed clear hypertrophy due to the high circulating TSH levels. (**B**) The body weight of primarily hypothyroid animals was reduced. However, hypothyroidism of central origin did not affect the body weight. (**C** and **D**) Neither body temperature nor the ratio of heart and body weight ratio was affected by hypothyroidism. $n = 10$ animals/group. Data are means ± standard error. $*p < 0.05$.

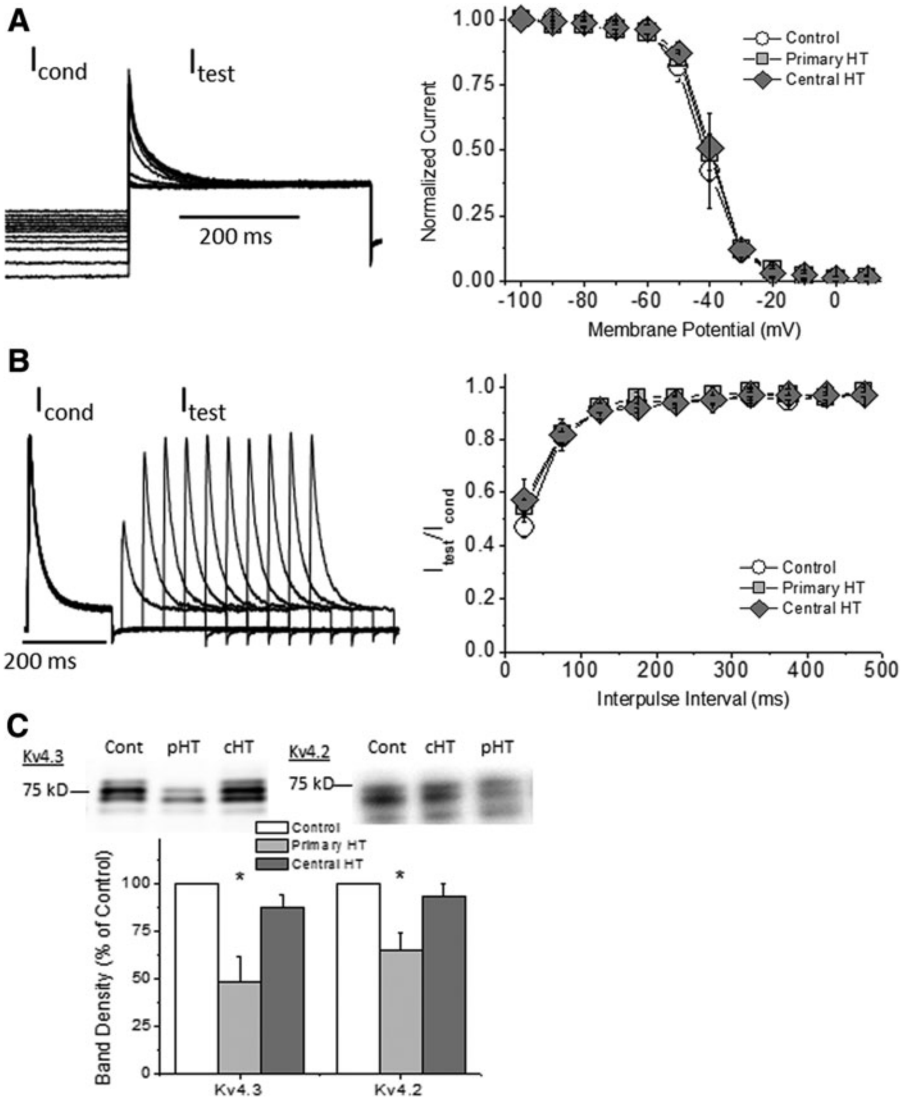

SUPPLEMENTARY FIG. S2. Transient outward K^+ current (I_{to}) recorded in ventricular cardiomyocytes from control and hypothyroid rats. Hypothyroidism did not modify either the voltage dependence of I_{to} inactivation (**A**) or the time dependence of the current recovery from inactivation (**B**). $n = 8$–10 cells from at least three animals. Data are means ± standard error. (**C**) Kv4.2 and Kv4.3 protein expression was reduced by primary (pHT) but not by central (cHT) hypothyroidism (Cont, control; $n = 3$ animals/group. $*p < 0.05$). Means ± SEM.

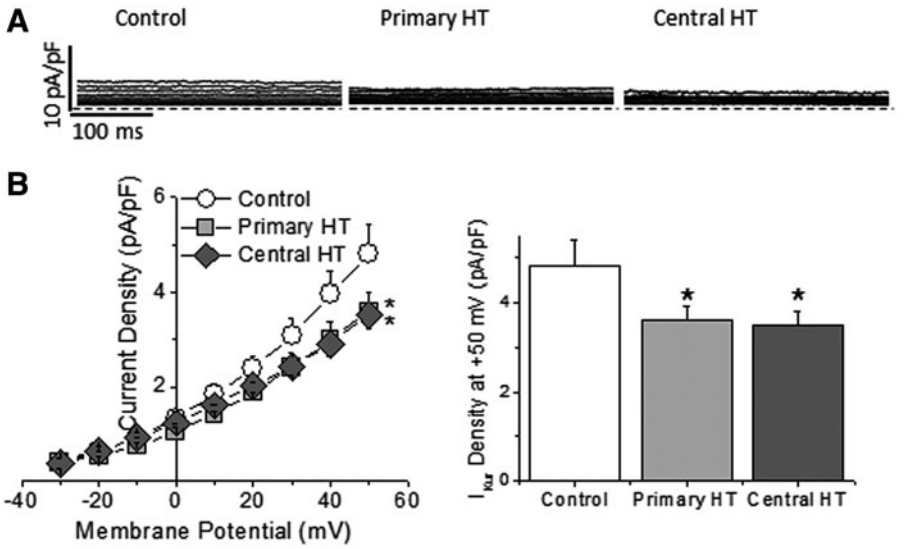

SUPPLEMENTARY FIG. S3. Ultra-rapid delayed rectifier K^+ current (I_{Kur}) recorded in ventricular cardiomyocytes from control and hypothyroid rats. (**A**) Traces of I_{Kur} recorded in ventricular myocytes isolated from control animals and from animals with primary and central hypothyroidism. Dashed lines represent the 0 current level. (**B**) Current–voltage relationships and maximum I_{Kur} amplitude at +50 mV, showing that both primary and central hypothyroidism reduced I_{Kur} density in a similar manner (n = 11–17 cells from at least three animals in each group; $^*p < 0.05$).

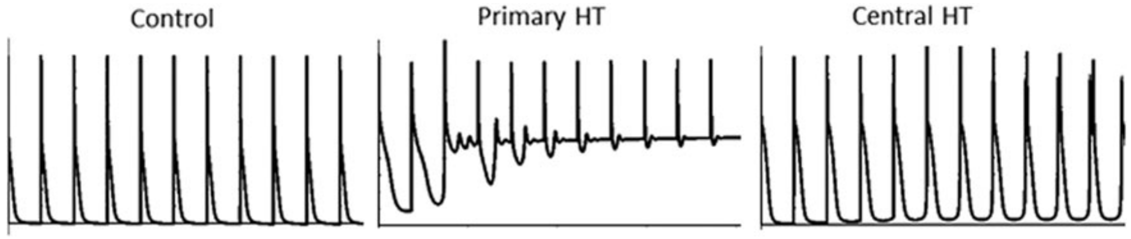

SUPPLEMENTARY FIG. S4. Effects of hypothyroidism on human atrial myocytes *in silico*. Computational models were generated to simulate human atrial action potentials. When the ionic conductances were changed to simulate the effects of primary hypothyroidism, arrhythmia appeared in resting conditions. When ionic conductances were adjusted to simulate central hypothyroidism, they do not develop arrhythmia, but some AP were accompanied by EADs.

Supplementary Table S1. Characteristics of the Patients Whose Atrial Appendage Samples Were Used in This Study

Patients (*n*)	8
Sex, male/female (*n*)	5/3
Body weight (kg)	81.1 ± 7.5 (59–123)
Height (cm)	164.5 ± 3.6 (155–181)
Age (years)	59 ± 2.2 (51–67)
Surgery (*n*)	
Valve	3
Revascularization	3
Combined	2

Data are presented as means ± standard error, followed by the minimum and maximum values in parentheses unless otherwise stated.

Supplementary Table S2. Forward and Reverse Primers for *KCND3*, *KCNH2*, *KCNQ1*, and *GAPDH*

Gene	*Forward 5′→3′*	*Reverse 5′→3′*
KCND3	TTA CTA CGA CGG GAG CAG CA	TCA CCC CAT TGT CTT TGT CC
KCNH2	TCG GAA GGG TTT GCA GTG	GAG TAC TTC CAG CAC GCC
KCNQ1	CAC CTG GAA GAT CTA CAT CCG	TCA CCC CAT TGT CTT TGT CC
GAPDH	ACC ATG GGG AAG GTG AAG GT	CAT GGG TGG AAT CAT ATT GG
